# Phylogenetic Distribution of Intron Positions in Alpha-Amylase Genes of Bilateria Suggests Numerous Gains and Losses

**DOI:** 10.1371/journal.pone.0019673

**Published:** 2011-05-17

**Authors:** Jean-Luc Da Lage, Frédérique Maczkowiak, Marie-Louise Cariou

**Affiliations:** 1 Laboratoire Evolution, génomes et spéciation, UPR 9034 CNRS, Gif sur Yvette, France; 2 Université Paris-Sud, Orsay, France; 3 Institut Curie, Université Paris-Sud, Orsay, France; University of Hong Kong, Hong Kong

## Abstract

Most eukaryotes have at least some genes interrupted by introns. While it is well accepted that introns were already present at moderate density in the last eukaryote common ancestor, the conspicuous diversity of intron density among genomes suggests a complex evolutionary history, with marked differences between phyla. The question of the rates of intron gains and loss in the course of evolution and factors influencing them remains controversial. We have investigated a single gene family, alpha-amylase, in 55 species covering a variety of animal phyla. Comparison of intron positions across phyla suggests a complex history, with a likely ancestral intronless gene undergoing frequent intron loss and gain, leading to extant intron/exon structures that are highly variable, even among species from the same phylum. Because introns are known to play no regulatory role in this gene and there is no alternative splicing, the structural differences may be interpreted more easily: intron positions, sizes, losses or gains may be more likely related to factors linked to splicing mechanisms and requirements, and to recognition of introns and exons, or to more extrinsic factors, such as life cycle and population size. We have shown that intron losses outnumbered gains in recent periods, but that “resets” of intron positions occurred at the origin of several phyla, including vertebrates. Rates of gain and loss appear to be positively correlated. No phase preference was found. We also found evidence for parallel gains and for intron sliding. Presence of introns at given positions was correlated to a strong protosplice consensus sequence AG/G, which was much weaker in the absence of intron. In contrast, recent intron insertions were not associated with a specific sequence. In animal *Amy* genes, population size and generation time seem to have played only minor roles in shaping gene structures.

## Introduction

Over thirty years ago, the discovery that eukaryotic genes were split, interrupted by non-coding DNA (e.g. [1], [2], [3]), caused a revolution in biology. The functional and evolutionary consequences of this unexpected gene structure were immediately foreseen by W. Gilbert in a famous and short article, in which he coined the terms intron and exon [4]. Since that time, the existence of introns in nuclear genes has been the source of a long-standing -and in some aspects, still lasting- debate. That debate concerns whether introns were present at the very beginning of life (the so-called “intron-early theory”), or were inserted much later in previously uninterrupted coding sequences (intron-late). Predictions of both theories have been tested using increasingly available data, although with sometimes opposite results. For instance, predictions of the intron-early theory regarding intron phase distribution (an excess of phase 0 introns) were confirmed for ancient genes, but contradictory interpretations were given by different authors, according to the analytical models they used [5], [6], [7]. For intron-late supporters, the age of the first introns was previously thought to be rather recent, given the initial lack of known introns in mitochondria-lacking eukaryotes (named Archezoa [8]). Much progress in the debate has been brought by the general effort of genome sequencing of a number of eukaryotes and prokaryotes, which has shown that (1) all sequenced prokaryotes lack spliceosomal introns and the elements of the splicing machinery, and (2) nearly all eukaryotes sequenced to date have at least a few spliceosomal introns, and all have elements of the spliceosome [9]. This demonstrates, according to many authors, that introns have been inserted in eukaryotes, at a very early stage of their evolution, so that all extant eukaryotes stem from an intron-bearing, and potentially intron-rich ancestor [10], [11], [12]. Potentially intron spread was tightly linked to the still mysterious origin of eukaryotes (e.g ref. [13]), maybe concomitant to it, and introns may have been a powerful booster of evolutionary novelties through exon-shuffling, alternative splicing, surveillance of mRNA integrity, promoting and favoring recombination [14], [15], [16], [17], [18]. Indeed, as Lynch and Richardson said [16], “it is likely that few, if any, of today's eukaryotes could survive without introns”.

The focus of the debate have shifted today to understanding intron gains and losses in the course of the history of eukaryotes, and estimating intron density in the genome of the last eukaryotic common ancestor. Are intron losses more frequent than intron gains, and if so, how long has this been the case? Or, in contrast, have gains been a high-frequency phenomenon at some time in the past, and thereafter have decreased to become rare events at more recent periods? Are rates of gains and losses correlated? Are rates of gains and losses lineage dependent? Genome data increasingly show significant levels of corresponding intron positions in conserved orthologous genes between remote eukaryotes, such as plants and animals [10], [19], [20], [21]. The extent to which these coincidental positions reflect true orthologous introns (i.e. present in the common ancestor) or parallel gains has been estimated by several workers, but remains controversial (reviewed in [22]).

A particular point that remains to be clarified is the gene-dependence of intron dynamics. Many authors have emphasized the importance of introns in the functions of a number of genes, for example because of the presence of regulatory information within introns (e.g. [23]), or because of the size of introns, which would simply act as timers for the proper temporal expression in the embryo development [24], or because of their role in mRNA surveillance through the nonsense mediated decay (NMD) process [17]. If introns differ significantly in their functional roles, then genomic studies of introns will tend to pool introns upon which selective forces act differently, and differences in functional fitness consequences of intron dynamics (i.e. gain or loss) will be overlooked.

Investigating intron dynamics in a single gene (or gene family) in various species may be valuable in this respect [25]. We compared intron-exon structures in alpha-amylase genes of animals, to illustrate the diversity of intron dynamics in genes with identical function in various organisms. Alpha-amylases form a multigene family in most organisms, including animals [26], [27], [28], [29], [30], but all copies have virtually the same function of degrading starch and related polysaccharides (http://www.cazy.org [31]). The amino acid sequence is poorly conserved among kingdoms [32], so that comparisons of intron-exon structures with plants or fungi are unreliable. Although it is possible to perform structural alignments owing to the conservation of the three-dimensional structure, such alignments remain ambiguous and limited to small parts of the sequence. Since studying intron gains and losses, through the conservation of intron positions, requires unambiguous alignments, we restricted our investigation to animal-type amylases in Bilateria, which align well to each others. No animal-type amylase (subfamily GH13_15/24 [33]) was found to date in non-bilaterian Metazoa, i.e. Porifera and Cnidaria [34] and Placozoa (unpublished). Instead, Fungus-type (GH13_1) alpha-amylase, also called *Dictyostelium*-type, seems to be the common and ancestral type in Unikonts, excluding Bilateria. Regarding the focus of the present work, it is interesting to note that the animal-type alpha-amylase studied here may be considered a “recent” gene, because it is assumed to be of bacterial origin through horizontal transfer [34]. Thus, the ancestral structure should have been intronless. As detailed below, the intron-exon structures are highly diverse among animal species, even, for example, within insects. Importantly, neither alternative splicing nor regulatory information within *Amy* introns have been reported to date in amylases. Thus, amylase genes will be considered free of this kind of constraints, so that the observed structural differences may be interpreted more easily: intron positions, sizes, losses or gains may be more likely related to factors linked to splicing mechanisms and requirements, and recognition of introns and exons, or to more extrinsic factors, such as life cycle and population size. Repeated intron losses in amylase genes have been reported in Drosophila and other Diptera [35], [36]. This study extends the investigation to the Bilateria.

## Materials and Methods

### Data collection

The animal species used in this study are listed in Table 1. To our knowledge, amylase genes are absent in the following bilaterian species whose genome has been deciphered: the louse *Pediculus humanus*, the flatworms *Schmidtea mediterranea* and *Schistosoma mansoni*, the leech *Helobdella robusta*, the aphid *Acyrtosiphon pisum*. Alpha-amylase genes were either determined experimentally or obtained from databases. In the latter case, the genome data, generally as draft releases, were searched with TBLASTN or BLASTP [37] using an animal sequence as a query. Given the similarity among animal amylases, we found that any animal *Amy* sequence could be used. For raw traces archives data, the best BLAST hits were assembled manually, with attention to the fact that several gene copies may occur. For experimental work, DNAs were extracted with classical methods. Polymerase chain reaction (PCR) amplifications were performed using combinations of a set of primers spanning a large part of the coding sequence, listed in Table S1. Amplification reactions were performed with increased elongation time to allow correct elongation, even in the presence of a further 1–2 kb of intronic DNA, in addition to the expected spliced length. For *Asterias rubens* (DNA supplied by A. van Wormhoudt, Station Marine de Concarneau), a partially amplified *Amy* gene was used as a probe for screening a mini-library The same method was applied to *Ceratitis capitata* and *Apis mellifera* (for the latter, a genomic library was kindly screened by M. Solignac in our laboratory). A genome walking strategy (Universal Genome Walking kit, Clontech) was applied to *Bibio marci*, *Musca domestica*, *Spodoptera frugiperda* (DNA from Sf9 cells), *Blaps mucronata*, *Periplaneta americana*, *Dysdera crocata*, *Lithobius forficatus*, *Corbicula fluminea*, *Cerastoderma edule*, *Mytilus edulis*, *Patella vulgata*.

**Table 1:** Alphabetical list of the species used in this study.

Species name	Source	Nr of gene copies*	Sequence length (bp)	Nr of introns	Accession or URL
*Acanthochitona fascicularis*	Lab	? ¶	3140¶	3	EU336959, EU336961, EU336963, EU336967, EU336969, EU336970
*Aedes aegypti*	DB	1	2325	1	AF000569
*Amphipholis squamata*	Lab	1	1413	1	EU336975
*Anopheles gambiae*	DB	1	2707	1	AAAB01008960, XM_316401
*Apis mellifera*	Lab	1	3038	4	AF259649
*Asphondylia sarothamni*	Lab	2	350; 624	1; 1	EU336971, EU336964
*Asterias rubens*	Lab	1	2574	2	AF286345
*Bibio marci*	Lab	2	1933; 2573	4; 2	AY082795, AY193771
*Blaps mucronata*	Lab	2	1634; 1339	3; 5	AF462603, AF468013
*Bombyx mori*	DB	1	5061	6	BAAB01056743; BAAB01084301; BAAB01023755
*Branchiostoma floridae*	DB, Lab	3	13121; 13095; 4316	8; 9; 9	http://genome.jgi-psf.org/ **
*Caenorhabditis briggsae*	DB	1	2520	7	http://www.sanger.ac.uk/cgi-bin/blast/submitblast/c_briggsae
*Caenorhabditis elegans*	DB	1	2547	6	Z81050
*Capitella teleta.*	DB	3	2549; 2320; 1675	6; 3; 3	http://genome.jgi-psf.org/ **
*Cerastoderma edule*	Lab	1	6868	4	EU336965
*Ceratitis capitata*	Lab	2	2067; 1652	2; 2	AF146757, AF146758
*Cimex lectularius*	Lab	1	848	3	EU336962
*Ciona intestinalis*	DB	1	4669	11	http://genome.jgi-psf.org/ **
*Ciona savignyi*	DB	1	5863	11	http://www.broad.mit.edu/annotation/ciona/
*Corbicula fluminea*	Lab	1	3957	4	AF468016
*Crassostrea gigas*	DB	1	4891	7	AF320688
*Daphnia pulex*	DB	4	3391; 2305; 3285; 3008	11; 11; 6; 14	http://wfleabase.org/blast/ http://genome.jgi-psf.org/ **
*Drosophila melanogaster*	Lab	2	1485; 1538	0; 1	X04569, AF022713
*Drosophila virilis*	DB	2	1544; 1526	1	U02029
*Dysdera crocata*	Lab	1	2947	2	EU336972
*Fugu rubripes*	DB	1	2711	8	http://fugu.biology.qmul.ac.uk/blast/
*Gallus gallus*	DB	1	4281	9	U63411
*Ixodes scapularis*	DB	? ¶	¶	5?	http://www.vectorbase.org/Tools/BLAST/
*Lepisma saccharina*	Lab	1	336	1	EU336968
*Leucophenga maculata*	Lab	1	1228	3	DQ021937
*Lingula anatina*	Lab	1	1204	1	EU336976
*Lithobius forficatus*	Lab	1	3628	3	EU336960
*Litopenaeus vannamei*	DB	1	3241	9	AJ133526
*Lottia gigantea*	DB	3	4119; 3247; >115 kb	5	http://traces.ensembl.org/; http://genome.jgi-psf.org/
*Megaselia scalaris*	Lab	1	1938	1	AF467104
*Musca domestica*	Lab	2	1635, 2038	2, 1	EF494036, EF494036
*Mytilus edulis*	Lab	1	4679	3	EU336958
*Nasonia vitripennis*	DB	3	2132; 2228; 2040	4; 5; 5	http://www.hgsc.bcm.tmc.edu/projects/nasonia/ **
*Oikopleura dioica*	DB	1	2419	1	Personal communication††, AJ496606
*Osmia cornuta*	Lab	1	1731	3	AF467103
*Ostrinia nubilalis*	DB	1	5980	6	U04223
*Patella vulgata*	Lab	1	2868	3	EU336973
*Pecten maximus*	DB, Lab	1	†	7	EU352806-EU352821
*Periplaneta americana*	Lab	1	905	2	EU336957
*Petromyzon marinus*	DB	1		9	http://traces.ensembl.org/; http://genome.ucsc.edu/
*Phascolosoma granulatum*	Lab	1	641	1	EU336966
*Pipunculidae* (unidentified)	Lab	1	1118	2	DQ021944
*Pyrrhosoma nymphula*	Lab	1	405	1	EU336974
*Saccoglossus kowalevskii*	DB	1	3149	2	http://traces.ensembl.org/
*Spodoptera frugiperda*	Lab	1	3795	6	AF280891
*Strongylocentrotus purpuratus*	DB	1	5493	3	NW_001464995 (130154–135667)
*Syrphidae* (unidentified)	Lab	1	1097	2	DQ021952
*Tetraodon nigroviridis*	DB	3	3859; 2619; 2623	8; 8; 8	AJ308233
*Tribolium castaneum*	DB	2; 4	5188; 11725	3;3; 4;4;4;4	U04271; http://www.ncbi.nlm.nih.gov/genome/guide/beetle/ **
*Xenopus tropicalis*	DB	2	6027; 10761	9; 9	http://genome.jgi-psf.org/ **

Lab: experimental data;

DB: data from public databases;

*: number of copies considered for this study;

¶ : sum of several fragments belonging to several copies;

† : several fragments generated using specific primers;

†† : personal communication of D. Chourrout and A. van Wormhoudt;

**: More detailed accession numbers may be found in Table S4.

### Intron identification

Putative introns were identified by translating the DNA sequences in the three frames and comparing the results with a manual alignment of known amylase proteins. Discrepancies and premature stops indicated the presence of introns, the boundaries of which were marked by finding donor and acceptor sites, which led to an in frame coding sequence. There were a few ambiguous cases in variable regions of the protein, where the alignment was uncertain. When possible, EST databases were used to check the predicted intron positions. Searches for remnants of transposable elements or duplicated neighboring exons within introns were performed using BLASTX (the introns of a single gene against Genbank, and against the coding sequence of the same gene, respectively). Intron losses or gains were inferred by parsimony, taking into account the phylogenetic relationships between the species harboring an intron at a given position. Introns were numbered, choosing intron 1 as the most widespread, conserved position among Bilateria.

## Results

### Number of introns, phylogenetic distribution

**Figure 1:**
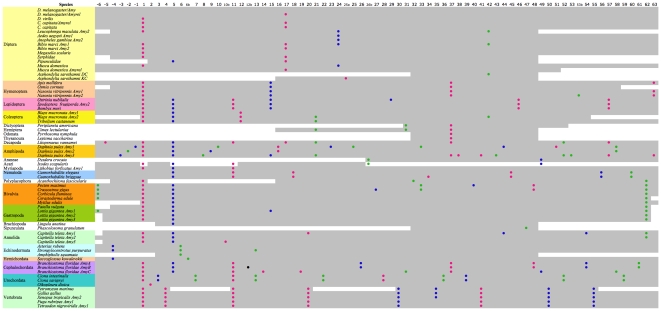
Distribution and nomenclature of introns along the amylase sequences for each gene and species of the sample. Sequence length available for each gene was shaded in grey. Introns were numbered according to their position, with intron 1 being the most widespread. When structures of tandem duplicated genes were similar (e.g. *Tetraodon nigroviridis*), only one was retained for the figure. Pink circles: phase zero introns; green circles: phase one introns; blue circles: phase two introns. The black circle in *B. floridae AmyB* indicates a dubious position, which was excluded from the study.

**Figure 2:**
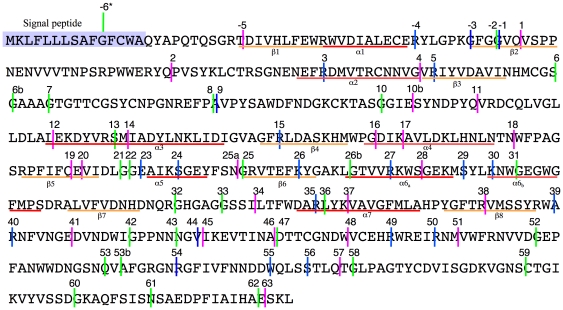
Positions of the identified intron on the typical pig amylase protein sequence (AF064742). Pink lines: phase zero introns; green lines: phase one introns; blue lines: phase two introns. The signal peptide is shaded. Secondary structures of the (β/α)_8_ barrel in the domain A of the protein are indicated as orange (β strands) or red (α helices). *: position −6 cannot be placed with accuracy, due to the high variability of the signal sequence (see text).

We obtained 79 genomic sequences of partial or complete amylase genes from 55 Bilaterian taxa. The extent of sequence available for each gene is shown on Figure 1. Seventy-four intron positions were found. We did not consider introns which may be found in the extra C-terminal domain present in some species (JLDL, unpublished). All the positions were mapped onto the pig pancreatic amylase protein sequence (Fig. 2). Figure S1 shows the mapping of intron positions onto a protein alignment, showing the conservation of the amylase sequence at each inferred position. Figure 1 shows the high diversity of intron-exon structures of *Amy* genes among animals: from no intron in *Drosophila melanogaster* to 14 introns in one gene of *Daphnia pulex*. In some documented cases, there is also a variation between gene copies of a single species: in *D. pulex*, we studied three copies, *Amy1*, *Amy2* and *Amy3*, with a total of 25 positions, 21 of which were specific of one of the three copies. In the Annelide *Capitella teleta*, three copies harbored a total of 7 intron positions, four of which were copy-specific. In the amphioxus *Branchiostoma floridae*, there were a total of 14 positions, among which 5 were found only in the *AmyC* copy. In contrast, in vertebrates, all the intron positions were shared by all gene copies within a species. It was for example the case in *Xenopus tropicalis*, *Tetraodon nigroviridis*, and human (not shown).

The diversity of intron/exon structures is highest among arthropods, with *D. pulex* and *D. melanogaster* being representative of the two extremes. The Lepidoptera seem to have many introns in their *Amy* genes, whereas Diptera have fewer. The case of Diptera *Amy* genes has been detailed in [36]. This high diversity may be not specific to arthropods, but our sample is biased; arthropods, especially insects, are largely represented here. More comprehensive data from other phyla might give similar results. For example, the tunicate *Ciona intestinalis* has numerous introns but not *Oïkopleura*, a distant Urochordate with a compact genome [38]. On the contrary, all vertebrates have many introns in their *Amy* genes, but to our knowledge, there is almost no variation in number: 8 in teleost fishes and 9 in other vertebrates.

### The case of tandemly arranged gene copies

As mentioned above, in some species, the same intron/exon structure is shared by the whole *Amy* gene family, whereas other species show striking differences between copies. The copies that share the same structure are generally physically close to each others, like in vertebrates. In the amphioxus, *AmyC*, which has several specific introns, is isolated, whereas the clustered *AmyA* and *AmyB* share most of their intron positions. Divergence in structure is correlated with divergence in sequence: *AmyA* and *AmyB* share 78% identity with each other at the protein level, but with *AmyC*, only 64% (A/C) or 55% (B/C). In the flour beetle *Tribolium castaneum*, four *Amy* copies are tandemly arranged, all of which have the same gene structure according to the genome annotation. Interestingly, in a previous cloning of two tandem copies [GenBank: U04271], both copies had been found to lack intron 5. In the whole genome sequenced, the four copies have this intron. This suggests a polymorphic presence/absence of this intron in *T. castaneum*, which has been rarely reported [39], [40], [41]. Another example is the wasp *Nasonia vitripennis*: two tandem copies *Amy2* and *Amy3*, very close to each other, share the same structure, whereas a third copy *Amy1*, found on a different contig, has a different structure. Thus, the conservation of intron/exon structure between gene copies, like the coding sequence, may be linked to their physical vicinity, as expected if the duplication events are more recent than with more distant copies. Additionally, there may be some concerted evolution (likely gene conversion was observed between *Amy1* and *Amy1'* of *Daphnia pulex*). However, although the intron positions are similar in tandem copies, it is also frequent that the sizes and sequences of homologous introns are very different (e.g. Amphioxus, Xenopus, Tetraodon). In the centipede *Lithobius forficatus*, there are two close tandem *Amy* copies. Intron 1 sequences (data not available for other introns in *Amy2*) are of different sizes (1417 bp vs. 1130 bp) and share no similarity at all. In the tandem *Amy* genes of *Nasonia vitripennis*, the coding sequences are 90% identical, whereas there is no similarity in introns, except the most distal (3′) intron 63, which is highly conserved.

### Widely conserved introns and phylum-specific introns

Figure 1 shows the intron positions for each of the sequences studied. It is immediately clear that only a few positions are widely shared among the sample: introns 1 and 11 are shared by various Protostomes and Deuterostomes; intron 5 may be also considered as widely shared if it is related to the vertebrate-specific intron 4 through intron sliding. All other intron positions are more restricted, but some are shared by several species of a single taxonomic entity. Two positions may be considered Protostome-specific (15 and 33); positions 17, 21, 37, 57, and 63 are specific to Arthropods. In addition, position 42 is insect-specific; position 12 is Coleoptera-specific; position 24 is Diptera-specific; position 46 is Lepidoptera-specific (moths only are represented). According to our data, mollusks-specific positions are scarce: position 62, which is also shared by the Annelide *Capitella*, and position −6, which is the only intron found in the region encoding the signal peptide. Owing to low sequence conservation of this region, orthology of this position among species is not sure (see Fig. 2). In Deuterostomes, only position 13 may be considered Deuterostome-specific, i.e. shared by several phyla. Six out of nine vertebrate intron positions are vertebrate-specific: introns 4 (assuming this does not represent an intron sliding event), 30, 35, 41, 50 and 55. Echinodermata are represented by a sea urchin, a sea star and an ophiura, which share positions −4 and 6. The Hemichordate *S. kowalevskii* shares with them position −4, and potentially its specific position 6b which could be misplaced due to ambiguous alignment. Hemichordates are the likely sister group of Echinodermata [42].

Clearly, these conclusions rely on the sampling, and could be changed with increasing data set. For instance, a number of positions are specific to *Caenorhabditis*, but other nematode species would be necessary to clarify the phylogenetic spread of these positions within Nematodes. More intron positions are bound to be found within most of the phyla represented here in the future. However, the collected data already allow to analyse some aspects of intron dynamics in *Amy* genes.

### Phases and insertion sites of introns

All *Amy* introns found so far are of the usual GT-AG type. Of the 74 intron positions, 25 are in phase zero (between two codons), 27 are in phase one (between the first et second base of a codon) and 22 are in phase two (between the second and third base of a codon). This distribution is not significantly different from a 1∶1∶1 proportion (χ^2^ = 0.513, ns), and is different from the usual bias observed toward phase 0 introns [6], [7]. We divided the sequence (Fig. 2) in five equal parts of 102 amino acids. The global intron distribution among these parts is not significantly uneven (χ^2^ = 5.09, ns). Intron-rich genes show a spread of the introns along the whole sequence. In contrast there is no clear trend for intron-poor genes (one or two introns) to have their introns located at the beginning of the sequence. However in some cases the pattern of distribution does appear unusual: in a number of Molluscan and Annelide sequences, most introns are concentrated in the 5′ part of the gene, with the exception of a single 3′ one.

**Figure 3:**
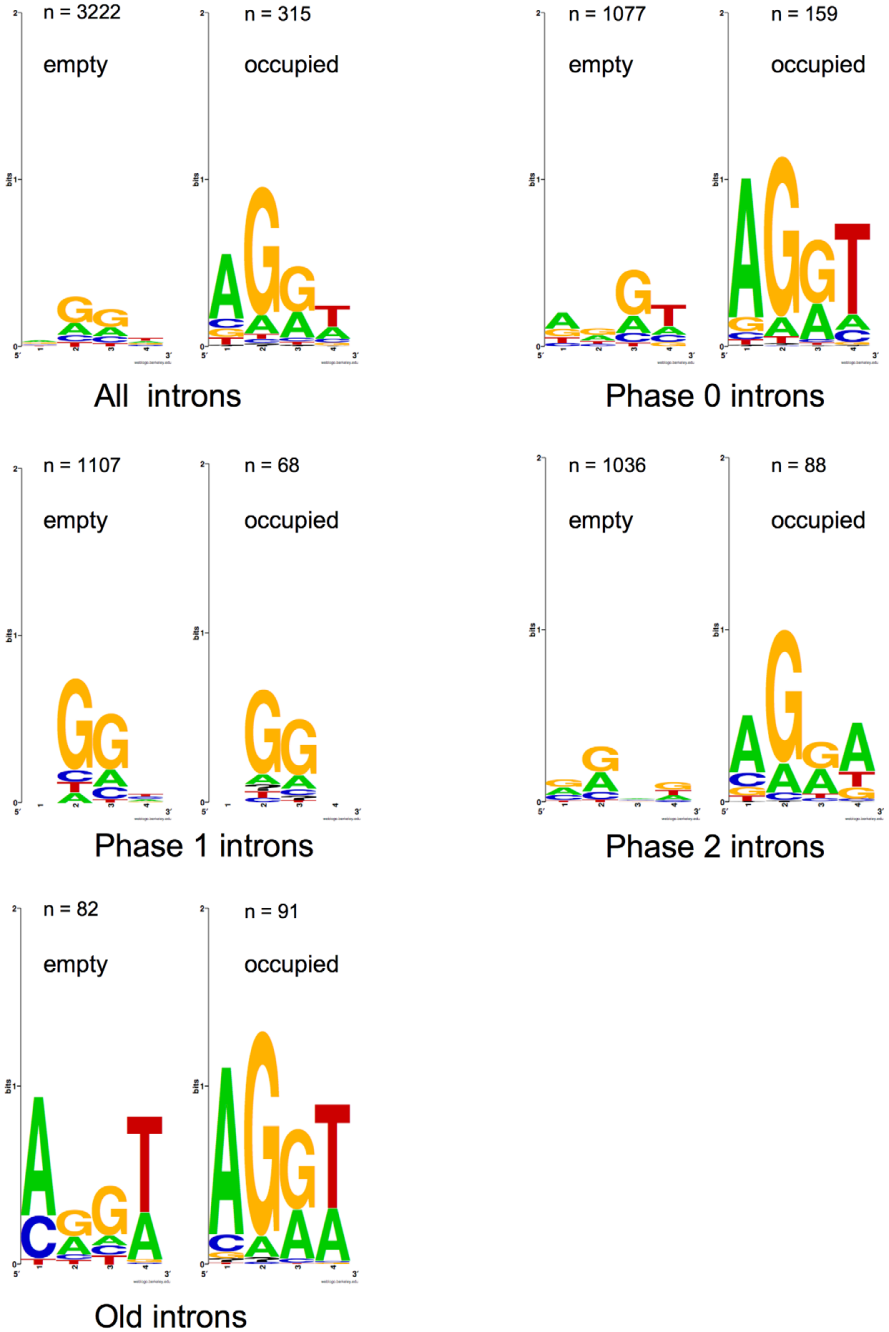
Conservation of the sequences surrounding intron positions in animal *Amy* genes. Positions −2, −1, +1 and +2 relative to the introns are shown. Intron positions in badly alignable regions were not used. Data are from Table S2. *n*: number of positions used; *occupied*: sites currently with an intron; *empty*: homologous sites devoid of intron. A: all intron positions; B: phase 0 positions; C: phase 1 positions; D: phase 2 positions; E: old positions, considered as ancestrally filled with an intron (pos. 1, 5, 11). Diagrams were made with Weblogo (http://weblogo.berkeley.edu/).

We also studied intron insertion sites. Exonic nucleotides flanking intron positions are not random: with observed preference for a consensus AG/(intron)/GT [43], the so-called “protosplice site”. To what extent this represents biases in the sites of intron creation, versus post-insertional selection, for instance for splicing efficiency, remains debated. For each intron position, we compared flanking exonic sequences across genes which did and did not contain an intron – “occupied” sites and “empty” sites (introns lost or never inserted) (Fig. 3 and Table S2). Occupied sites showed a stronger protosplice consensus, except for phase 1 introns, for which a G/G minimal consensus was common to occupied and empty sites, perhaps because they were inside conserved glycine codons. For phase 0 introns, the classical protosplice consensus AG/GT was found when introns were present. This sequence was also particularly clear for the three presumably oldest sites, i.e. positions 1, 5, 11, for which an absence of intron, supposed to be unambiguously due to a loss, was related to a weaker consensus (Fig. 3E). This suggests a relaxation of constraints after intron loss at these positions. We also checked for a few “recently gained” intron positions (25a, 27, 29, 53b pooled) whether the surrounding sequence showed a consensus motif. There was no consensus when the intron was absent, assumed to be the ancestral state (232 occurrences, not shown), nor in the four cases of insertion (Table S2). In these few cases, insertion was not linked to the presence of a preferential sequence. These results are consistent with the notion that the positions of the *de novo* intron insertions are largely unbiased, and that selection drives the emergence of protosplice-like sequences following intron insertion [44]; in addition, the finding that protosplice sites are observed for very old introns but not for much younger ones, may suggest that the transition to protosplice sites is a slow process.

### Intron losses and gains; ancestral and recent introns

Investigating the rates or frequencies of intron losses and gains in *Amy* genes is the main goal of this study. Relating the presence/absence factual data to the loss/gain interpretation is mainly a matter of sampling. From our data set, we have been able to identify numerous intron losses at various periods in the past, ancient losses basal to a whole phylum, or more recent, phylogenetically more limited losses. For example, Figure 1 shows that intron 1, the most common one, is certainly ancestral in Bilateria, and has been lost several times, in some dipteran genes, in Echinoderms, in the tunicate *Oïkopleura* and in *Caenorhabditis*. We focused on Drosophila *Amy* genes more thoroughly by PCR and we confirmed that intron 1 was lost independently in several lineages within the last 30 million years, but only in the subgenus *Sophophora*, in which the number of gene copies is more than one (excluding the old paralog *Amyrel*), whereas no intron loss was recorded in the subgenus *Drosophila*, in which *Amy* seems to be generally single-copy. It is possible that increased intron losses could be related to the number of gene copies. Intron position 11 is also considered ancestral, but would have been lost, according to the data, in Mollusks, in Echinoderms, and in Diptera, Coleoptera and Hymenoptera. Intron 5 is also widespread and old, since it is shared throughout Protostomes. It is also present in the amphioxus, but since it is absent from other Deuterostomes, it is not clear whether it is an ancestral position or represents a case of parallel insertion, unless one considers intron 4 (vertebrates) as the result of a 5 bp-sliding from position 5, a hypothesis for which there is no evidence.

**Figure 4:**
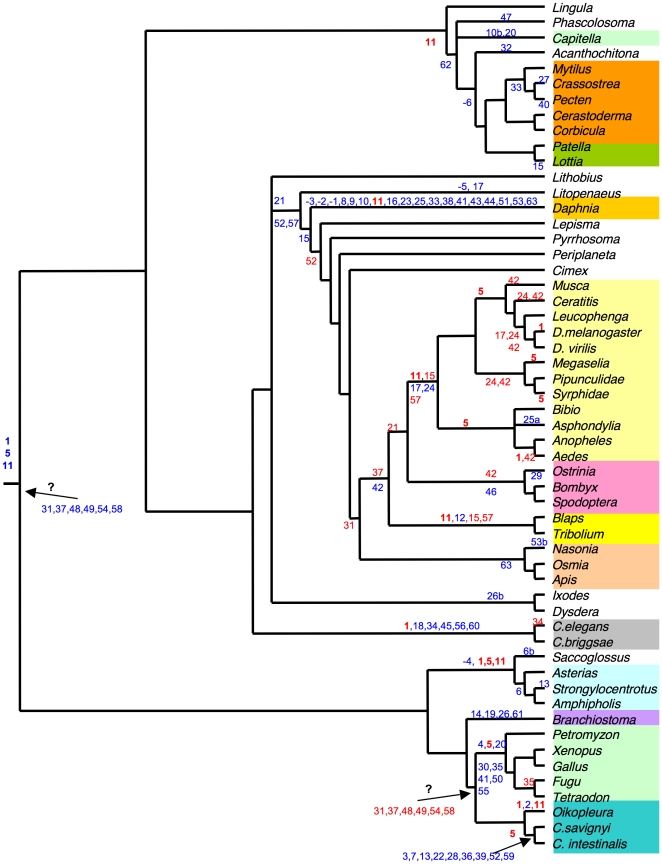
A scenario for intron gains and losses along the bilaterian phylogeny. Phylogenetic tree of the Bilateria species included in this study, consensus drawn from refs. [42], [110], [111], [112], [113], [114], [115]. Intron positions were placed at nodes corresponding to the putative losses (red numbers) or gains (blue numbers), deduced from the phylogenetic distribution of Fig. 1. Alternative possibilities are proposed on Table S3. For clarity of the figure, the repeated losses in Protostomes of the positions shared with Amphioxus (question mark) were not reported. Color code for taxonomical groups is the same as in Fig. 1.

The tree in Figure 4 summarizes the proposed status of lost or gained introns, according to their phylogenetic distribution. Inferences of gains or losses on the tree reflect parsimony reconstructions; other interpretations are plausible for a number of positions (Table S3) because the data suggest either massive losses or parallel gains, which is a common issue in this kind of investigations. For instance, in Figure 4, introns 15 and 33, which are Protostome-specific, have been considered each as cases of independent gains. The Deuterostome-specific intron 13 was considered as two independent gains rather than ancestral to Deuterostomes. As another example, position 20 is shared by vertebrates and one gene of the worm *Capitella*. This is more suggestive of parallel gains. Indeed, some of these uncertain cases could be solved with an increased sample. Some cases are however clear: intron 35 has been lost in fishes since it is present in the lamprey *P. marinus* and the tetrapods; intron 34 has been lost in *C. elegans*, since it is present in *C. briggsae*, *C. brenneri*, *C. remanei* and *Pristionchus pacificus* (not shown). Other examples may be found in Figure 1.

Cases of gains are also likely, but most often less directly conclusive, and there is no obvious case of “recent” gain. The seemingly high number of gains on terminal branches of the tree (Fig. 4) does not mean that these gains are truly recent. Intron gain cases were inferred when the phylogenetic distribution of the intron was scarce or limited to a clade, given a correct sampling in related taxa or phyla (see paragraph *Widely conserved introns and phylum-specific introns* above). The assignment of intron losses and gains is thus dependent on sampling. The extreme case is for introns limited to one species or one gene in the data set. For instance, one can infer an intron gain for position 25a in the Nematocere *A. sarothamni* because the dipteran sampling is good; intron 53b may have been gained in the wasp *N. vitripennis*, by comparison with other Hymenoptera sequences. In Bivalves, the oyster and scallop sequences each show a specific intron (27 and 40, respectively), which could be examples of species-specific intron gains. However, this interpretation is weakened because we do not know all *Amy* copies of each species. It is thus possible that these introns are present in other copies of the other species. The most striking example of possible gains is that of *Daphnia pulex*. This species has the most split *Amy* gene (14 introns), and 14 species-specific positions, which can be considered gained. Indeed, many likely paralog-specific gains are inferred. Considering *Amy1* and *Amy2*, which are related to each other, since they are clustered in an animal *Amy* gene tree (not shown), there are only two common positions (not shared with *Amy3* nor any other sequence), but six additional positions which are unique to *Amy1*, reflecting either gains in *Amy1* or losses in *Amy2*. The structure of *Amy3* is less derived, sharing five introns with other Arthropods: 15, 21, 37, 52, 57, 63. This suggests a complex structural history after gene duplications in this lineage. However, though we are confident that these positions are gains, the sampling for Branchiopoda is limited to this species, so that the exact antiquity of these introns is unknown. They could also be shared by other, unsampled Crustacean clades. Overall, we did not find conclusive cases of intron gains more recent than 150–200 million years.

In contrast, phylum-specific positions, corresponding to ancient gains stemming from the origin of the phyla, perhaps during the Cambrian or late Precambrian explosion, seem to be frequent. It is visually clear on Figure 1 for Deuterostomes, but also in Protostomes. In contrast, there are few positions shared by several Protostome phyla, or by several Deuterostome phyla. Regarding Deuterostomes, at most one intron position is shared exclusively by them (and it may be a parallel gain). Surprisingly, in the amphioxus, 7 of 13 positions are shared with Protostomes, but not with other Deuterostomes (5, 31, 37, 48, 49, 54, 58).

### Intron sliding

Intron sliding is suspected to have occurred when two intron positions are very close to each other between two gene copies or two species. The phylogenetic sample must be sufficient enough to rule out parallel insertions. In our study, some positions are conspicuous: in *D. pulex*, positions −2 and −1, one base pair apart, harbored by two paralogs *Amy2* and *Amy1*, respectively, create each with intron 1, a very small exon of 8 bp and 7 bp, respectively. This was confirmed by ESTs (http://wfleabase.org/genomics/est/) and our experimental resequencing of this gene region. Introns 8 (*Amy3*) and 9 (*Amy2*) are also one base pair apart. Rather than independent gains in paralogous copies, both cases strongly suggest 1 bp intron sliding, the most frequently encountered, according to some authors [20], [45]. However, the intron sequences are very different, so that the ultimate evidence, intron sequence similarity, is lacking. Other putative candidates for intron sliding may be introns 44–45, or 46–47 (Fig. 2) but the phylogenetic distribution of these positions is rather in favor of independent gains. The case of introns 4–5 was mentioned above, but cannot be solved with our data.

## Discussion

The origin of animal amylases remains enigmatic. Up to now, animal-type alpha-amylases (i.e. GH13_15/24 [33]) were found only in bilaterian metazoa. The age and the origin of the ancestral bilaterian amylase are not well established (discussed in ref. [34]). Recent estimates suggest the origin of Bilateria to be rather close to the basal Cambrian [46], [47], [48]. We have proposed that *Amy* arose by horizontal transfer from a bacterium after the split of Cnidaria [34]. This implies a massive and not so old colonization by introns. Basu et al. [49] have shown that genes transferred to the nucleus from the plastid precursor cyanobacterium were quickly colonized by introns. Nuclear genes of mitochondrial origin were also shown to be colonized quickly [50]. But these events are probably much older. Importantly, the bilaterian *Amy* genes cannot be regarded as “ancient genes” such as those included in clusters of orthologous genes (COG) and used in comparative genomic studies (e.g. [7], [10], [19], [21], [51], [52]).

The simple observation of the variety of structures among the holometabolous insects was an invitation to try reconstituting the history of intron movements, not only in insect amylases, but also in other animals. Such data could help understanding more general rules of intron dynamics. Focusing on this single gene, either as a single-copy or, most often in animals, duplicated, we have identified dozens of intron positions. It is clear that new positions will still be found by searching in taxonomic groups not studied yet.

An important result of this study is that few intron positions can be identified as certainly ancestral, but it does not mean that the last bilaterian common ancestor had only these relic introns. On the other hand, numerous positions may stem from the origins of individual phyla or sub-phyla, in the late Proterozoic or during the Cambrian, and be concomitant to the genome novelties that accompanied new bauplans (what Babenko et al. [53] called “transitional periods of evolutionary history”). The ancestral *Amy* structure would have been partly reset in most phyla. After a burst of intron movements, especially gains, basal to Bilateria, losses would have become predominant until now, at various rates. In *Amy* genes, intron gains and losses seem to have occurred in a temporally irregular manner. The uneven nature of these rates has been already reported in comparative genomic studies [51], [53], [54]. This picture is akin to the one depicted in genomic studies [22], [53] or in single gene studies [55], [56]. A majority of comparative genomic studies have suggested excess of losses over gains over the last 500 MY (e.g [49], [57], [58], [59], except in Fungi [60]. However, increasing data suggest that intron acquisition is still ongoing (reviewed in [61]). Case studies have often shown frequent intron losses too, e.g. [35], [36], [39], [62], [63], [64], [65], [66], [67]. But in some cases, where the sample was phylogenetically large with known divergence dates, intron gains were found to be dominant [68].

### Intron resets in Deuterostomes and the amphioxus conundrum

At the genome level, vertebrates share many more intron positions than expected with the sea anemone *Nematostella vectensis*, a non-bilaterian animal [69] and also with the polychaete annelid *Platynereis dumerilii* in a genome fragment of 30 contiguous genes [70] (we did not confirm this in the Polychaeta *Capitella teleta*). This suggests that vertebrates could have conserved ancestral exon-intron structures, while most other phyla would show derived patterns. However, this reasoning does not hold regarding *Amy* since we posit that the bilaterian *Amy* gene originated from bacteria, and then was devoid of introns in the early Bilateria. Genome data indicate that 85% of intron positions in the amphioxus *B. floridae* are shared with vertebrates [71]. Thus it was unexpected to find that seven positions out of 13 in the amphioxus *Amy* genes were shared with Protostomes, and with Protostomes only, whereas few intron positions are shared among Deuterostomes. This situation is intriguing. This suggests either repeated parallel gains, or retention of ancestral positions. Parallel gains are possible: other Deuterostomes share about one *Amy* intron position with Protostomes. But seven parallel gains in this single species raise questions. On the other hand, retention of ancestral positions implies that the last ancestor between Protostomes and Deuterostomes had *Amy* gene(s) with a Protostome-like structure, which, in Deuterostomes, was retained solely in the amphioxus; but there is no clear pattern of conservation of the amphioxus introns with those of a subset of Protostomes. Instead, these coincident positions are scattered among various protostome species. Many losses are needed for this scenario. Thus, there is still little evidence for this hypothesis. A mixed model is also likely. In any hypothesis, the observed pattern implies numerous intron losses and gains in vertebrates, urochordates, echinoderms. In many genes of the urochordate *Oïkopleura dioica*, introns have strikingly moved even after the split from *Ciona intestinalis*, so that most of them are species-specific [72], [73]. More generally, the numerous losses and gains basal to most phyla, which are necessary to explain the observed patterns, suggest a positive correlation between the rates of gain and loss.

### Patterns and mechanisms of intron gains and losses in amylase genes

A good phylogenetic coverage is crucial for inferring true gains. In this respect, our best data are for insects. Recent cases of losses and gains have been documented in the genus *Drosophila*, showing that losses were eight times as frequent as gains [74]. Accordingly, we have identified several independent losses of the same intron in *Amy* genes from various Drosophila species probably less than 20 MY ago [35], [36], [75], [76]. Regarding gains, the most recent datable gains in insect amylases are not younger than 200 MY, given the divergence times and origin of the holometabolous orders [77]. The gain of intron 25a in *A. sarothamni* may be younger, but it is not possible to date it. Insect *Amy* genes have rather undergone mostly intron losses (Fig. 4).

In contradiction with Qiu et al. [7], who assume constant rates for a given gene across all the phyla, intron dynamics in *Amy* genes has been quite different among the studied lineages, e.g. arthropods *vs.* vertebrates: in vertebrates, almost no intron movement occurred since the split of lampreys and jawed vertebrates 500 MY ago [78]. We noticed only one loss in teleost fishes. This low variability in intron positions is considered typical of vertebrate genomes [79], [80], although recent works suggest that changes occurred in some gene families [81]. During the same period of time, and even shorter regarding insects, arthropods amylases evolved a wealth on intron-exon structures. What is the origin of such a diversity? What are the intrinsic or extrinsic factors involved in intron losses and gains and their fixation?

The most frequently assumed mechanism of intron loss considers a cDNA intermediate produced by endogenous reverse transcriptases [51], [74], [79], [82], [83]. The cDNA is most often a truncated, 3′ part of the spliced gene. Thus, 3′ biased losses are expected, and intron richness should be higher by the 5′ part of the gene [51], [84]. Our data seem to be roughly in agreement with such a mechanism, in that there are no widely shared introns in the 3′ half of the *Amy* genes, suggesting that recurrent losses may have restricted most 3′ introns to particular clades. However, “orphan introns” (those found only in one species or in a restricted group) also frequently exist in the 5′ part of the gene, and in several species, a proximal, clearly old intron was lost (e.g. intron 1), but not other, more distal ones. This shows that the mechanism may be more complex, or use more 5′ partial retrotranscripts, with internal priming. In our study, genomic deletions seem not to be responsible for the observed losses, since all intron losses are accurate, removing or adding no coding sequence, contrary to what occurred for example in the *jingwei* gene of *Drosophila teissieri* [40] or in pufferfishes [80].

To be transmitted to the progeny, an intron loss or gain must occur in the germline, as pointed out earlier [63], [67], [85]. In our context of a retrotranscript intermediate, this implies that *Amy* genes experiencing intron losses should be transcribed in the germline, at least at a basal level. Differential levels of germline expression among species could account for the differential variability of intron-exon structure. For example, to explain the intron-rich structures of *Amy* genes, with little variability in the Lepidoptera studied here, since their divergence from each other over 75 MY ago (dates from fossil records [77]), as compared to Diptera, we may hypothesize an absence of germline transcription. Unfortunately, there are little data about *Amy* expression in the germline for the species studied here, except for *D. melanogaster*, *C. elegans* and mammals (GEO profiles: www.ncbi.nlm.nih.gov/sites/entrez?db=geo; flyatlas.org). Nonetheless, supporting this hypothesis, the comparison between *Amy* and its paralog *Amyrel* within flies shows that while the *Amy* intron (intron 1) was lost several times, there was almost no case of loss in *Amyrel* (intron 17) in more than 200 species studied, diverged from 0.5 to over 80 MY [36], [86]. The Flyatlas data suggest indeed that in *D. melanogaster*, *Amy* is transcribed in testis whereas *Amyrel* is not (nor in ovaries).

Another factor may influence the final intron-exon pattern and intron dynamics. The efficiency of the splicing machinery to splice out introns is linked to its ability to recognize and identify exons or introns properly. The exon definition model [87] suggests strong constraints on the size of exons in vertebrates, with a critical upper size, beyond which an exon could be misrecognized and skipped. This implies that intron losses (and also sliding, if mediated by a loss and gain mechanism [88], [89]) would be often counterselected. This would explain the low variability in intron-exon structure of vertebrate genes, including *Amy*. This model might apply to other species with short exons and long introns of our sample, such as Lepidoptera or the shrimp *L. vannamei*. In some other species, in contrast, introns are numerous but very short, e.g. in *D. pulex*, *C. elegans* and *C. briggsae*. In these species, the genome of which has been sequenced, short introns are a general feature (wfleabase.org; [90]). This could be linked to mechanistic requirement for proper intron recognition [91]. Some species harbor *Amy* genes with both short and long introns, short and long exons, probably requiring mixed splicing recognition mechanisms. *Daphnia pulex* deserves particular mention for its 7 bp and 8 bp exons. Such small exons are rare in animals [92], [93], [94], [95]. Their correct splicing might require strenghtened splicing sites and splice enhancers [56], or else they could be skipped. The corresponding introns −2 and −1 are bounded by GCG/GA and CGG/TG, respectively, which are not strong, canonical protosplices, but unknown signals may lie inside the introns surrounding these short exons.

A correlation between the intron number or length and the level of expression of amylase could be expected as an optimization to lower the cost of transcription in highly expressed genes [96], [97]. But in many Drosophila species, amylase is highly expressed, irrespective of intron presence and size (no intron in some species, one intron longer than 1,2 kb in *D. phalerata*). In mammals, nine long introns are present, yet amylase is produced at a high level. In *A. thaliana*, Knowles et al. [98] also found no relationship between intron gain or loss and gene expression.

Intron richness of organisms may be influenced by generation time and cell cycle duration, as mentioned and discussed earlier [38], [54], [72], [99]. This view assumes that fast reproducing animals would have less and/or shorter introns, as a genome compaction. This has been examplified in the urochordate *Oïkopleura dioica*, whose life cycle is four days long [72]. However, our data on amylase genes seem not to show a clearcut discrimination such as long-cycle animals with many introns *vs.* short-cycle animals with few introns. For instance, *C. elegans* probably has dozens of generations a year, and a six-intron *Amy* gene (although introns are short). The same observation applies to the moth *S. frugiperda*, which has about 12 generation per year in the wild, and yet, its amylase gene has six long introns, increasing the gene size by 150%.

The influence of population size on shaping gene structures has been proposed as a major evolutionary force [16], [100], [101]. It is assumed that many introns, considered slightly deleterious, could have been fixed by genetic drift in the putatively small populations occurring at the early times of eukaryote evolution, explaining that many unicellular eukaryotes, with large population sizes, experienced intron losses driven by purifying selection, whereas multicellular organisms with small population sizes retained their introns, and then remained intron-rich. Such effects may be difficult to test in Metazoa, owing to the order of magnitude of the differences in population sizes between species, which is not as high as between unicellular and multicellular organisms. However, it is likely that effective population sizes are comparable, for instance, between moths and flies, which have very different *Amy* gene structures. This suggests that demographic factors had little influence in our case.

Several mechanisms of intron gains have been proposed. But since intron sequences evolve rapidly, the origin and the insertion mechanism of an intron cannot be identified, unless the intron gain is recent enough. Indeed, we never found the origin of gained introns in our data. We found no case of introns created by insertion of transposable elements, although a few introns contained transposon fragments (Table S5). However, retrotransposable elements may act through the synthesis of reverse transcriptase (RT) by *Pol* genes. As for intron loss, RT would produce partial cDNAs, which would be reinserted in the genome. Internal duplications at the DNA level could also be a source of introns if correct splicing sequences were present at the duplicate ends. We found neither trace of such intronization of duplicated exons in the putatively most recently gained introns nor evidence of intron gain through (recent) intron transposition between two genes or within the same gene by reverse splicing. Our ability to detect intron transposition may be scrambled (see [99], [102]) because sequence similarity may be shared by different introns without any direct relationship. For example, in the moth *S. frugiperda*, a part of the intron 5 of one *Amy* copy was a repeated element, which was also found in introns of other genes in various Lepidoptera (not shown). Recent works suggest that DNA repair through nonhomologous end joining could generate introns from any template, explaining that one rarely discover a “parental” sequence. This mechanism creates short direct repeats at the insertion site [61]. This was found in Daphnia for very recent introns [103]. However, these repeats may diverge quickly as time goes on, so that our data are unable to show such traces.

The protosplice is defined as a preferential target sequence for insertion, involving the spliceosome machinery [43]. Such sequences have been shown to be “active” as potential targets for intron insertion [104], [105]. Insertions could alternatively occur at random, with subsequent elimination of inserts located in an environment not suitable for proper splicing, or adaptation of the surrounding sequences to improve splicing efficiency; this hypothesis has been infirmed by [52]. Our data show a global preference for *Amy* introns to be surrounded by sequences matching the conservative protosplice sequence AG/G; interestingly, the surrounding bases −2 to +2 fitted the protosplice far less well in “empty” sites (Fig. 3, Table S2). Oldest introns (positions 1, 5, 11) showed a good fit to the sequence AG/GT, whereas we found gained intron positions for which the surrounding sequence was completely different (Table S2). This contrasts with ref. [20], [44] who suggested that old introns are surrounded by sequences deviating from the protosplice consensus, contrary to more recent introns. It may be due to the shorter time scale of our study.

The multigene nature of *Amy* genes should not be omitted in this discussion, since it has been proposed that intron gains are more frequent in paralogous genes, partly due to relaxation of selective constraints on the duplicates ([53], discussed in ref. [106]). The conspicuous case of *D. pulex* could illustrate this trend. On the other hand, we also observed more losses in Drosophila *Amy* genes, in species that had several gene copies. At a rather short time scale, significant numbers of gains and losses were found between paralogous genes in *A. thaliana* [98]. In addition, it has been suggested that, in the case of tandemly arranged genes, introns tend to diverge in length and sequence to prevent illegitimate recombination [72]. We have observed such a trend for example in vertebrates, in the amphioxus, in *L. forficatus*, in *N. vitripennis*.

In this single gene study, we have shown that contrasted intron patterns occur even in the absence of selection for informational content in introns or alternative splicing, but might depend on mechanistic requirements. Our data suggest that intron dynamics is a various and changing story, which depends on both the lineage, even at an intra-phylum scale, and the gene considered, and also probably depends on the intron position. Hence, we share the wise conclusion of Jeffares et al. [54]. Additional complete genomes covering much better the eukaryotes will increasingly enable to draw a much more correct estimate of intron dynamics at a broader time scale. In addition, comparative genomics of related species, such as the 12 Drosophila genomes [107], [108], [109], and even at the intraspecific level (ref. [103] shows an extraordinary snapshot of ongoing intron gains in waterfleas) bring valuable data at a small time scale, which is the best way of estimating current rates and mechanisms of intron gain and loss.

## Supporting Information

Figure S1 **Mapping of intron positions on a protein alignment of animal alpha-amylases performed using CLUSTALW** [**116**] **and manually adjusted for additional sequences.** Intron positions were mapped on top of the figure, except position −6. Color code: pink lines: phase zero introns; green lines: phase one introns; blue lines: phase two introns. Amino acid colors are RasMol default colors. The alignment was edited with Geneious v.5.3.6.
(TIF)

Table S1 **List of the the most useful PCR primers used for partial amplification of alpha-amylase genes in animals.** Position is relative to the *D. melanogaster* sequence. *: specific for Drosophila *Amy* gene amplification excluding the *Amyrel* paralog.
(DOC)

Table S2 **Sequences surrounding intron sites. Color code is as in Figure 1.** Black: no intron. For unalignable positions, no corresponding intronless site could be indicated in other genes (e.g. position 10).
(XLS)

Table S3 **Alternative scenarii to the intron gains/losses shown on Figure 3.**
(DOC)

Table S4 **Accessions of *Amy* genes in sequenced genomes.**
(DOC)

Table S5 **Results of BLASTX searches in long introns of the data set. RT: reverse transcriptase.**
(DOC)
