# Supplementary material for: Phylogenetic Distribution of Intron Positions in Alpha-Amylase Genes of Bilateria Suggests Numerous Gains and Losses
Source: PLoS One. 2011 May 17;6(5):e19673. doi: 10.1371/journal.pone.0019673 (PMC3096672; doi:10.1371/journal.pone.0019673)
Supplement: Table S1 — List of the the most useful PCR primers used for partial amplification of alpha-amylase genes in animals. Position is relative to the D. melanogaster sequence. *: specific for Drosophila Amy gene amplification excluding the Amyrel paralog. (DOC) [file pone.0019673.s002.doc]

**Supplementary Table S1**: List of the the most useful PCR primers used for partial amplification of alpha-amylase genes in animals. Position is relative to the *D. melanogaster* sequence. *: specific for Drosophila *Amy* gene amplification excluding the *Amyrel* paralog.

**Primer name sequence position sense**

1U+ GTNCAYCTNTTYGARTGG 94-11 fwd

FEWKW* CAYCTNTTYGARTGGAARTGG 97-117 fwd

12u+ TTYGARTGGAARTGGDVNGAYATHGC 103-128 fwd

WWERYQP GGTGGGARCGTTAYCARCC 221-239 fwd

2U+ GGYTGRTANCNYTCCCACCA 220-239 rev

KHM GAYGCNNNNAARCAYATGTGGC 610-631 fwd

HSDSIGK* TTNCCRATGGAGTCGGARTG 796-815 rev

DNHD GAYAAYCAYGAYAAYCARCG 907-926 fwd

CEHREV TGNCKCCANCGRTGYTCRCA 1144-1163 rev

1230+ TTGCTGCCRTTRTCCCACCA 1219-1238 rev

REV1400 ACRTCRCARTANGTNCCNGC 1330-1349 rev

1400+ CCNGADATNAYRTCRCARTA 1339-1358 rev
