# Supplementary material for: Phylogenetic Distribution of Intron Positions in Alpha-Amylase Genes of Bilateria Suggests Numerous Gains and Losses
Source: PLoS One. 2011 May 17;6(5):e19673. doi: 10.1371/journal.pone.0019673 (PMC3096672; doi:10.1371/journal.pone.0019673)
Supplement: Table S3 — Alternative scenarii to the intron gains/losses shown on Figure 3. (DOC) [file pone.0019673.s004.doc]

**Supplementary Table S3**: Alternative scenarii to the intron gains/losses shown on Figure 3

**Intron position alternative scenario**

13 Unique gain in Deuterostomes + multiple losses

15 Unique gain in Protostomes + multiple losses

17 Unique gain in Pancrustacea + multiple losses

20 Ancestral in Bilateria + multiple losses

31, 37, 48, Parallel gains in Amphioxus and

49, 54, 58 various Protostomes

41 Ancestral in Bilateria + multiple losses

63 Unique gain in Pancrustacea + multiple losses
