# Supplementary material for: Phylogenetic Distribution of Intron Positions in Alpha-Amylase Genes of Bilateria Suggests Numerous Gains and Losses
Source: PLoS One. 2011 May 17;6(5):e19673. doi: 10.1371/journal.pone.0019673 (PMC3096672; doi:10.1371/journal.pone.0019673)
Supplement: Table S4 — Accessions of Amy genes in sequenced genomes. (DOC) [file pone.0019673.s005.doc]

**Supplementary Table S4:**

**accessions of *Amy* genes in genomes at the Joint Genome Institute**

http://genome.jgi-psf.org/

*Daphnia pulex* Amy1: Scaffold 51;227854-230577

*Daphnia pulex* Amy1': Scaffold 51:221021-223435

*Daphnia pulex* Amy2: Scaffold 183:118848-121831

*Daphnia pulex* Amy3: Scaffold 21:55237-57930

*Capitella teleta* Amy1: Capca1/scaffold_695:54667-57359

*Capitella teleta* Amy2: Capca1/scaffold_327:160841-163442

*Capitella teleta* Amy3: Capca1/scaffold_94:92081-93755

*Xenopus tropicalis* Amy1: Xentr4/scaffold_415:387118-402937

*Xenopus tropicalis* Amy2: Xentr4/scaffold_415:406036-412186

*Branchiostoma floridae* AmyA: Brafl1/scaffold_71:1376853-1384692

*Branchiostoma floridae* AmyB: Brafl1/scaffold_71:1416971-1430194

*Branchiostoma floridae* AmyC: Brafl1/scaffold_426:616661-621517

*Lottia gigantea* Amy1: Lotgi1/sca_3:1216127-1219857

*Lottia gigantea* Amy2: Lotgi1/sca_30: 1239815-1243133

*Lottia gigantea* Amy3: Lotgi1/sca_13: 2367743-2406992, Lotgi1/sca_13:2482832-2483041

*Ciona intestinalis*: Cioin2/chr_05q:958902-964196

**accessions of *Amy* genes in genomes at the Human Genome Sequencing Center**

http://www.hgsc.bcm.tmc.edu/projects/

*Nasonia vitripennis* Amy 1: Contig 21 (NW_001815904) 2893046-2895177

*Nasonia vitripennis* Amy 2: Contig 22 (NW_001816015) 1263747-1261520

*Nasonia vitripennis* Amy 3: Contig 22 (NW_001816015) 1259933-1257892

**accessions of *Amy* genes in genomes at the NCBI**

http://www.ncbi.nlm.nih.gov/genome/guide/beetle/

*Tribolium castaneum* Amy 1: NW_001092775.1|TcaLG2_WGA10_1:3145365-3147044

*Tribolium castaneum* Amy 2: NW_001092775.1|TcaLG2_WGA10_1:3149533-3151271

*Tribolium castaneum* Amy 3: NW_001092775.1|TcaLG2_WGA10_1:3152474-3154215

*Tribolium castaneum* Amy 4: NW_001092775.1|TcaLG2_WGA10_1:3155346-3157090
