# Supplementary material for: Phylogenetic Distribution of Intron Positions in Alpha-Amylase Genes of Bilateria Suggests Numerous Gains and Losses
Source: PLoS One. 2011 May 17;6(5):e19673. doi: 10.1371/journal.pone.0019673 (PMC3096672; doi:10.1371/journal.pone.0019673)
Supplement: Table S5 — Results of BLASTX searches in long introns of the data set. RT: reverse transcriptase. (DOC) [file pone.0019673.s006.doc]

**Supplementary Table S5:** Results of BLASTX searches in long introns of the data set. RT: reverse transcriptase

| Species | Intron position | Hit length | Score/  E-value | accession | Hit identification |
| --- | --- | --- | --- | --- | --- |
| *Ciona intestinalis* | 39, 59 | 110 bp, 99 bp | 50.1/ 8e-04 | CAB01920 | similarity to IG superfamily protein *Ciona* |
| *Ostrinia nubilalis* | 29 | 137 bp | 47.8/ 0.007 | ABO45231 | RT *O. nubilalis* |
| *T. nigroviridis Amy1* | 41 | 296 bp | 153/ 6e-35 | AF355375 | putative RT *T. rubripes* |
| *Cerastoderma edule* | 62 | 889 bp | 89/ 7e-25 | XP_780253 | similar to CR1 *D. rerio* 2 RT *S. purpuratus* |
| *L. forficatus Amy2* | 5 | 788 pb | 111/ 2e-22 | XP_001337779 | similar to pol polyprotein *D. rerio* |
| *X. tropicalis Amy1* | 4 | 89 bp | 45.4/ 0.077 | AAA49022 | similar to pol-like *Gallus gallus* |
| *B. floridae AmyB* | 48 | 689 bp | 197/ 2e-47 | XP_001186727 | similar to endonuclease-RT *S. purpuratus* |
